# Dramatic morphological changes in liposomes induced by peptide nanofibers reversibly polymerized and depolymerized by the photoisomerization of spiropyran

**DOI:** 10.3389/fmolb.2023.1137885

**Published:** 2023-03-30

**Authors:** Yingbing Liang, Shigesaburo Ogawa, Hiroshi Inaba, Kazunori Matsuura

**Affiliations:** ^1^ Department of Chemistry and Biotechnology, Graduate School of Engineering Tottori University Koyama-Minami 4-101, Tottori, Japan; ^2^ Centre for Research on Green Sustainable Chemistry Tottori University Koyama-Minami 4-101, Tottori, Japan

**Keywords:** peptide nanofiber, liposome, photoisomerization, self-assembly, polymerization/depolymerization, spiropyran, merocyanine, artificial cytoskeleton

## Abstract

Cytoskeletons such as microtubules and actin filaments are natural protein assemblies, which dynamically control cellular morphology by reversible polymerization/depolymerization. Recently, the control of polymerization/depolymerization of fibrous protein/peptide assemblies by external stimuli has attracted significant attention. However, as far as we know, the creation of an “artificial cytoskeleton” that reversibly controls the polymerization/depolymerization of peptide nanofiber in giant unilamellar vesicles (GUVs) has not been reported. Here, we developed peptide nanofiber self-assembled from spiropyran (SP)-modified *β*-sheet-forming peptides, which can be reversibly polymerized/depolymerized by light. The reversible photoisomerization of the SP-modified peptide (FKFEC^SP^KFE) to the merocyanine-peptide (FKFEC^MC^KFE) by ultraviolet (UV) and visible light irradiation was confirmed by UV–visible spectroscopy. Confocal laser scanning microscopy with thioflavin T staining and transmission electron microscopy of the peptides showed that the SP-peptide formed *β*-sheet nanofibers, whereas the photoisomerization to the merocyanine-peptide almost completely dissociated the nanofibers. The merocyanine peptide was encapsulated in spherical GUVs comprising of phospholipids as artificial cell models. Interestingly, the morphology of GUV encapsulating the merocyanine-peptide dramatically changed into worm-like vesicles by the photoisomerization to the SP-modified peptide, and then reversibly changed into spherical GUV by the photoisomerization to the MC-modified peptide. These dynamic morphological changes in GUVs by light can be applied as components of a molecular robot with artificially controlled cellular functions.

## Introduction

Cytoskeletons in eukaryotic cells such as microtubules, actin filaments, and intermediate filaments are fibrous protein assemblies, which spatiotemporally polymerize and depolymerize to control cell morphology and intracellular/extracellular movements (Pederson and Aebi, 2002; Wade, 2009; Eldirany et al., 2021). The dynamic polymerization/depolymerization of the cytoskeleton plays important roles in the deformation and movement of various membrane systems and the arrangement of cell organelles, cell division, muscle contraction, and ciliary movement (Luna and Hitt, 1992; Ramaekers et al., 2004; Hall, 2009; Fletcher and Mullins, 2010). The polymerization and depolymerization of microtubules and actin filaments are dynamically controlled by the hydrolysis of guanosine triphosphate (GTP) and adenosine triphosphate (ATP), respectively. It is challenging to control the dynamics of the polymerization and depolymerization of the cytoskeletons using artificial materials.

Giant unilamellar vesicles (GUVs) with diameters near cell size are widely utilized in research as cellular models for the physicochemical understanding of processes involving biological lipid membranes (Lasic and Papahadjopoulos, 1995). Recently, it was reported that the polymerization/depolymerization of cytoskeletons and physical perturbation to GUVs induced deformation in GUVs, as in natural cells (Sekine et al., 2012; Shi and Baumgart, 2015; Sato et al., 2017; Steinkühler et al., 2020; Ganar et al., 2021). For example, Tanaka et al. (2018) reported that the adjustment of osmotic pressure and cleavage of actin filaments with gelsolin induced reversible deformation in GUVs between the spindle and spherical morphologies. Koseki and Suzuki (2020) successfully constructed neuron-like vesicles by osmotic shrinking in GUVs. Li et al. (2021) developed self-driven artificial cells encapsulating active mitochondria that can generate ATP and demonstrated reversible morphological changes in GUVs with the growth of actin filaments by the generated ATP. However, there were problems such as the irreversibility of GUV deformation (Loiseau et al., 2016), long response time (Fanalista et al., 2018), and small morphology change (Liu et al., 2021).

Recently, the polymerization/depolymerization of various peptide/protein fibers has been controlled by external stimuli, such as enzymatic reactions (Haines, et al., 2005; Tanaka, et al., 2015; Chen, et al., 2016; West, et al., 2018), light (Bosques and Imperiali, 2003; Doran, et al., 2014; Nakamura, et al., 2021), and redox (D’Souza, et al., 2022; Yao, et al., 2021; Bowerman and Nilsson, 2010). For example, Pires et al. (2015) successfully formed peptide nanofibers on the cell surface by enzymatic dephosphorylation to induce cell death. Sendai et al. (2013) reported the light-induced reversible polymerization/depolymerization of protein nanotubes comprising GroEL (a natural molecular chaperone) modified with photochromic spiropyran (SP). Bashirzadeh et al. (2020) recently reported the light-controlled reversible formation/dissociation of hydrogels self-assembled from SP-modified short peptide, Fmoc-KK^SP^KF-NH_2_, by the photoisomerization of SP to protonated merocyanine (MCH^+^). However, to the best of our knowledge, there is no example of the dynamic control of GUV deformation by reversibly controlled polymerization/depolymerization of self-assembling peptide nanofibers mimicking natural cytoskeletons. Major challenge of this study is to develop a self-assembling material that can reversibly and dramatically deform GUVs by polymerization/depolymerization upon external stimuli.

Previously, we pioneered a photoinduced peptide nanofiber growth system by conjugating a *β*-sheet-forming peptide (FKFEFKFE) with DNA (dA_20_), an assembly inhibitory site, using a photodissociative amino acid (Furutani et al., 2015). The equipping of this system with the GUV and nucleosphere (DNA microassembly) promotes motility driven by light-induced nanofiber formation (Inaba, et al., 2018; Inaba, et al., 2021). Considering that the light-induced spatiotemporal control of nanofiber growth is irreversible, the reversible control of the polymerization/depolymerization mimicking of natural cytoskeletons has not been realized.

**FIGURE 1:**
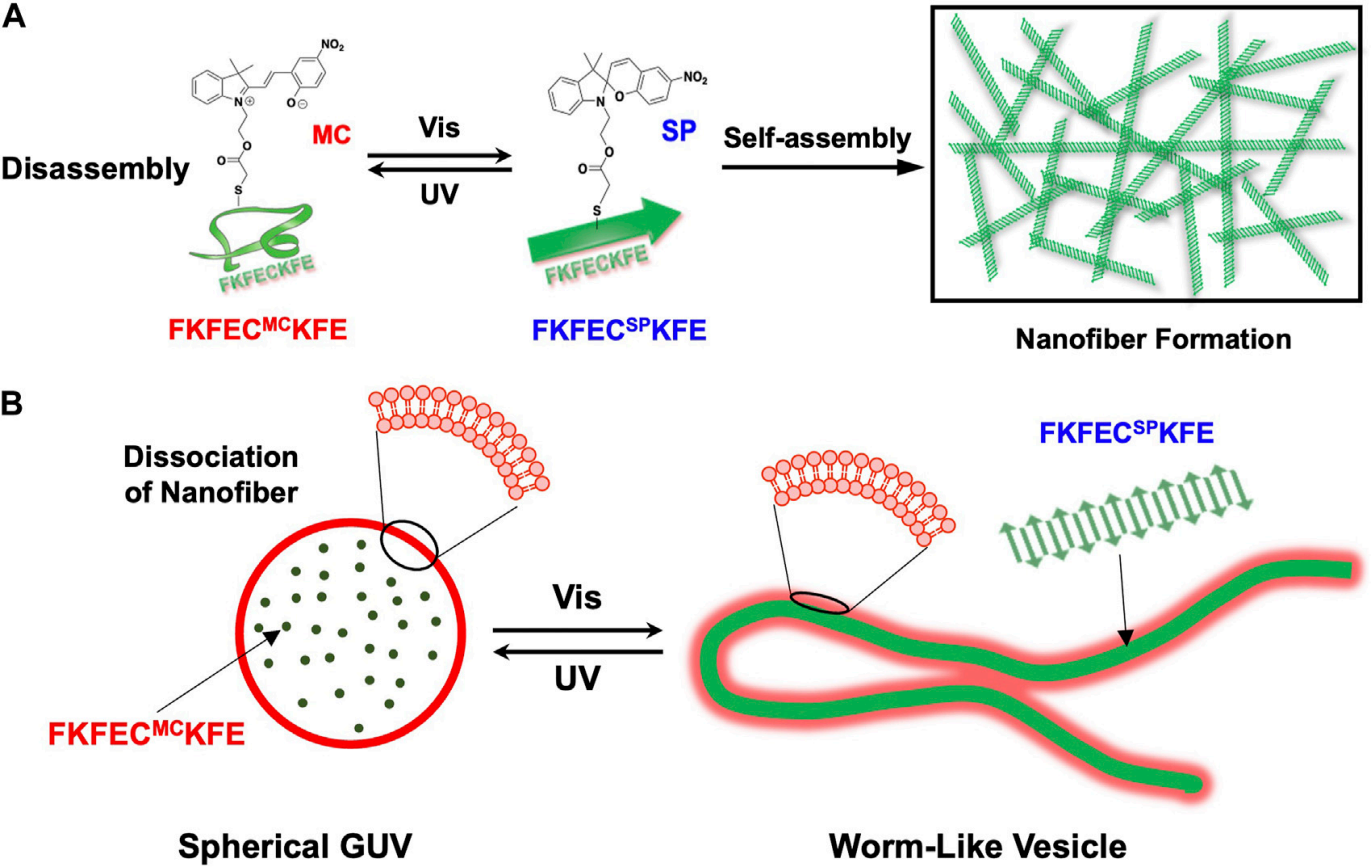
**(A)** Schematic of reversible nanofiber formation and dissociation of FKFEC^SP/MC^KFE peptides by photoisomerization. **(B)** Schematic of the reversible and dramatic morphological changes that occurred after the photoisomerization of FKFEC^SP/MC^KFE in the giant unilamellar vesicle (GUV).

Here, to create artificial cytoskeletons that dynamically control the deformation of GUVs by the light-induced reversible polymerization/depolymerization of peptide nanofibers, we designed a *β*-sheet-forming peptide modified with a photochromic dye, SP (Figure 1A). Uncharged SP with a dipole moment of 4.3 D was isomerized by ultraviolet (UV) light irradiation to zwitterionic merocyanine (MC) with a dipole moment of 17.7 D (Klajn, 2014). The photoisomerization of SP/MC with changes in the structure and dipole moment can be harnessed for the reversible control of the polymerization/depolymerization of peptide/protein assemblies (Sendai et al., 2013; Bashirzadeh et al., 2020). We demonstrated the reversible control of the polymerization/depolymerization of peptide nanofibers self-assembled from the SP/MC-modified *β*-sheet-forming peptide (Figure 1A) and dramatic morphological changes in the GUVs encapsulating the peptide by light irradiation (Figure 1B). By creating a photo-responsive artificial cytoskeleton that mimics the dynamic control of the eukaryotic cell cytoskeleton, the dramatic deformation of the GUV through the polymerization and depolymerization of peptide nanofibers by light irradiation was achieved. From the perspective of molecular robotics reconstructed from artificial molecules, it is proposed here that polymerization and depolymerization of peptide nanofibers is another mechanism that enables the dynamic properties of living cells.

## Materials and methods

### General

Ultrapure water with high resistivity (>18 MΩ cm) purified using the Millipore Purification System (Milli-Q water) was used as a solvent for the peptides. Reagents were obtained from a commercial source and used without further purification. Reversed-phase high-performance liquid chromatography (RP-HPLC) was performed at ambient temperature using a Shimadzu LC-6AD system equipped with a UV–visible detector [220 and 280 nm, Shimadzu SPD-10 A(V) vp] and Inertsil ODS-3 (GL Science) columns (250 × 4.6 or 250 × 20 mm). Matrix-assisted laser desorption/ionization time-of-flight mass spectrometry (MALDI-TOF MS) was conducted using an UltrafleXtreme instrument (Bruker Daltonics) in the linear/positive mode with α-cyano-4-hydroxy cinnamic acid (α-CHCA) as the matrix. Circular dichroism (CD) spectra were recorded at 25°C in a 1.0 cm quartz cell using a Jasco J-820 spectrophotometer equipped with a Peltier-type thermostatic cell holder.

### Synthesis of *β*-sheet-forming peptide, FKFECKFE

*β*-Sheet-forming peptide bearing Cys (FKFECKFE) was synthesized on Fmoc-Glu (OtBu)-Alko resin (543 mg, 0.125 mmol/g; Watanabe Chemical Ind. Ltd.,) by fluorenylmethyloxycarbonyl (Fmoc)-based coupling reactions (4 equiv. of Fmoc amino acids). An *N*-methyl pyrrolidone (NMP) solution containing (1-cyano-2-ethoxy-oxoethylidenaminooxy) dimethyl amino-morpholino-carbenium hexafluorophosphate (COMU, 4 equiv.) and *N, N*-diisopropylethylamine (DIPEA, 8 equiv.) was used as the coupling reagent. Fmoc deprotection was achieved by adding 2 mL of a solution of piperidine/*N, N*-dimethylformamide (DMF) = 40/60 (vol) to the column and stirring for 3 min, then removing the solution. Next, 2 mL of a solution of piperidine/DMF = 20/80 (vol) was added and stirred for 10 min. After removing the solution, the resin was washed five times with NMP. The progression of the coupling reaction and Fmoc deprotection was confirmed using a 2,4,6-trinitrobenzene sulfonic acid (TNBS) test kit (TCI Co. Ltd.,). The peptidyl resins were washed with NMP and dried under a vacuum. The peptide was deprotected and cleaved from the resin through treatment with a cocktail of trifluoroacetic acid (TFA)/1,2-ethanedithiol/triisopropylsilane/thioanisole/water at a ratio of 8.25/0.25/0.1/0.5/0.5 (mL) at room temperature for 4 h. The reaction mixture was filtered to remove the resins, and the filtrates were concentrated under vacuum conditions. The peptide was precipitated by adding methyl *tert*-butyl ether (MTBE) to the residue, and the supernatant was decanted. After washing thrice with MTBE, the crude product was purified by RP-HPLC, eluting with a linear gradient of CH_3_CN/water containing 0.1% TFA (5/95 to 50 min). The fraction containing the desired peptide was lyophilized to afford 2.34 mg of a flocculent solid (45.1% yield), confirmed by MALDI-TOF MS (matrix, α-CHCA; *m/z* = 1077 [M]^+^).

### Synthesis of bromoacetyl spiropyran

Here, 1-(2-hydroxyethyl)-3,3-dimethylindolino-6′-nitrobenzopyrylospiran (251.0 mg, 0.71 mmol) and 4-dimethylaminopyridine (137.5 mg, 1.12 mmol) were placed in an eggplant flask and purged with N_2_ gas. The mixture was dissolved in dry toluene (6 mL), and bromoacetyl bromide (100 μL, 1.07 mmol) was added dropwise at 25°C with stirring. The reaction mixture was stirred for 2 h at 25°C under a nitrogen atmosphere and quenched by adding water (10 mL). The mixture was extracted with dichloromethane (30 mL) and washed thrice with water. The organic layer was dried over anhydrous MgSO_4_ overnight, and the MgSO_4_ was removed by filtration. The filtrate was concentrated under reduced pressure to obtain a reddish-brown solid. Subsequently, the product was purified by silica-gel column chromatography using dichloromethane as the eluent. After the evaporation of dichloromethane under reduced pressure, 269 mg (80% yield) of bromoacetyl spiropyran was obtained as a yellow-brown powder. MALDI-TOF MS: *m/z* found: 472.3 ([SP-^79^Br]^+^), calcd. 472.1, m*/z* found: 474.3 ([SP-^81^Br]^+^), calcd. 474.1.^1^H NMR (500 MHz, CDCl_3_, Supplementary Figure S8): *δ*/ppm; 8.01–8.03 (2H, m); 7.22 (1H, t); 7.10 (1H, d); 6.90–6.94 (2H, m); 6.75 (1H, d); 6.67 (1H, d); 5.93 (1H, d); 4.32 (2H, s); 3.77 (2H, t); 3.50 (2H, q); 1.16–1.2 (6H, s).

### Synthesis of spiropyran-modified peptide FKFEC^SP^KFE

FKFECKFE peptide (3.53 mg, 3.28 μmol) was added to a solution of bromoacetyl spiropyran (2.2 mg, 4.59 μmol) in DMF (700 μL) and DIPEA (100 μL), and the mixture was stirred for 4 h at 4°C. The product was purified by RP-HPLC, eluting with a linear gradient of CH_3_CN/water containing 0.1% TFA (5/95 to 50 min). The fraction containing the FKFEC^SP^KFE peptide was lyophilized to afford 1.33 mg of flocculent solid (27.6% yield), confirmed by MALDI-TOF MS (matrix, α-CHCA; *m/z* = 1469 [M]^+^).

### Photoisomerization of FKFEC^SP/MC^KFE

FKFEC^SP^KFE peptide (100 μM) was dissolved in a 10-mM phosphate buffer (pH, 7.4) and incubated for 30 min at 60°C, followed by incubation for 10 min at 25°C. The peptide solution was irradiated with UV light (365 nm, 5 mW, and 5 cm) using a Jasco FP-8200 spectrofluorometer for 10–180 min at 25°C, and UV-visible spectra were recorded using a Jasco V-630 spectrophotometer. Afterward, the peptide solution was irradiated with visible light (580 nm, 5 mW, and 5 cm) for 10–180 min at 25°C, and UV-visible spectra were recorded.

### Circular dichroism spectra of FKFEC^SP/MC^KFE

A solution of FKFEC^SP^KFE peptide (100 μM) in a 10-mM phosphate buffer (pH, 7.4) was incubated at 60°C for 30 min and left in the dark at 25°C for 24 h. The CD spectrum of the peptide solution was measured at 25°C in a 1.0-cm quartz cell using a Jasco J-820 spectrophotometer. After UV light irradiation of the solution (365 nm, 5 mW, and 5 cm) for 80 min using the Jasco FP-8200 spectrofluorometer, followed by incubation in the dark at 25°C for 24 h, the CD spectrum of the FKFEC^MC^KFE peptide solution was recorded at 25°C.

### Fluorescence spectra of thioflavin T

A solution of thioflavin T (100 μM) and FKFEC^SP^KFE peptide (100 μM) in a 10-mM phosphate buffer (pH, 7.4) was incubated at 60 °C for 30 min and left in the dark at 25°C for 24 h. The fluorescence spectrum of the solution was recorded using a Jasco FP-8200 spectrofluorometer at 25°C at an excitation wavelength of 450 nm. After the UV light irradiation of the solution (365 nm, 5 mW, and 5 cm) for 80 min using a Jasco FP-8200 spectrofluorometer, followed by incubation for 24 h in the dark at 25°C, the fluorescence spectrum of the solution was recorded. The fluorescence spectra of the FKFEC^SP^KFE peptide (100 μM) alone and thioflavin T (100 μM) alone in a 10-mM phosphate buffer were recorded separately under the same condition.

### Transmission electron microscopy (TEM)

Carbon-coated Cu grids (thin carbon film TEM grids; Alliance Biosystems) were hydrophilized by plasma treatment (25°C, 60 Hz, 500 VA, 40 s, JEOL HD Treatment). Aliquots (5 μL) of the aqueous sample solutions were applied to the hydrophilized carbon-coated Cu grids for 1 min and removed using filter paper. Subsequently, the TEM grids were instilled in a staining solution, 2% phosphotungstic acid (Na_3_(PW_12_O_40_) (H_2_O)_n_) (5 µL), for 2 min and removed using filter paper. After the sample-loaded Cu grids were dried *in vacuo*, they were observed by TEM (JEOL JEM 1400 Plus) at an accelerating voltage of 80 kV.

### Preparation of giant unilamellar vesicle-encapsulating peptide

Here, 1-palmitoyl-2-oleoyl phosphatidylcholine (POPC, Funakoshi Co., Ltd.) and Atto 550-labeled 1,2-dioleoyl-*sn*-glycero-3-phosphoethanolamine (Atto 550-DOPE, Funakoshi Co., Ltd.) with a molar ratio of 200:1 were mixed with D-glucose (1 equiv. of the lipids) in chloroform/methanol (2/1, v/v). The solution was poured into a glass test tube and dried under reduced pressure overnight. A 10 μM FKFEC^SP^KFE peptide solution in a 10-mM phosphate buffer (pH, 7.4) containing 100 μM thioflavin T was incubated at 60°C for 30 min, followed by 10 min at 25°C. Thereafter, the peptide solution was irradiated with UV light (365 nm, 5 mW, and 5 cm) using the Jasco FP-8200 spectrofluorometer for 80 min and incubated for 24 h at 25°C in the dark. Thereafter, the dried lipid film was hydrated with the peptide solution (100 μL) in a 10-mM phosphate buffer (pH, 7.4) containing 100 μM thioflavin T at 25°C and incubated for 1 h in the dark at 25°C. The final concentration was [POPC] = 1 mM [Atto 550 DOPE] = 5 μM [FKFEC^SP/MC^KFE] = 10 μM [thioflavin T] = 100 μM.

### Confocal laser scanning microscopy (CLSM)

The thioflavin T-stained FKFEC^SP^KFE, FKFEC^MC^KFE peptides, and GUVs encapsulating peptides were observed by CLSM using a FluoView FV10i (Olympus). The FKFEC^SP^KFE peptide solution (100 μM) in a 10-mM phosphate buffer (pH, 7.4) containing 100 μM thioflavin T was incubated at 60 °C for 30 min, followed by 10 min at 25°C. CLSM was performed by placing the prepared peptide solution of FKFEC^SP^KFE in a glass bottom dish. Thereafter, the peptide solution was irradiated with UV light (5 mW, 5 cm) for 80 min using the Jasco FP-8200 spectrofluorometer at 365 nm and incubated for 24 h at 25°C in the dark. Afterward, the CLSM of the peptide solution of FKFEC^MC^KFE (100 μM) in a 10-mM phosphate buffer (pH, 7.4) containing 100 μM thioflavin T was performed.

The dispersion of GUV encapsulating FKFEC^MC^KFE peptide (2 μL) was dropped onto a glass slide and covered with a cover glass, and CLSM was performed. The dispersions of GUV encapsulating FKFEC^MC^KFE peptide on the glass slide were irradiated with visible light at 505 nm (CL-1503, Asahi Spectra, 52 mW, 10 cm) for 3 min, and images were obtained every 10 s in the CLSM time-lapse mode, followed by 1 h of continuous video recording. The dispersions of GUV encapsulating FKFEC^SP^KFE peptide on the glass slide were irradiated with UV light at 365 nm (CL-1503, Asahi Spectra, 30 mW, 10 cm) for 3 min, and images were obtained every 10 s in the CLSM time-lapse mode, followed by 1 h of continuous video recording.

The final concentration was [POPC] = 1 mM [Atto 550 DOPE] = 5 μM [FKFEC^SP/MC^KFE] = 10 μM [thioflavin T] = 100 μM. Thioflavin T was excited at 491 nm and observed through a 508-nm emission band-pass filter (green). Atto 550 and merocyanine was excited at 553 nm and observed through a 577-nm emission band-pass filter (red).

## Results

### Synthesis of spiropyran-modified *β*-sheet-forming peptide

To create nanofibers that reversibly polymerize and depolymerize upon photoisomerization, we designed a *β*-sheet-forming peptide with SP (Figure 1). The central Phe residue of the *β*-sheet-forming peptide, FKFEFKFE (Marini et al., 2002), which forms nanofibers based on antiparallel *β*-sheets, was substituted with Cys to modify SP. The FKFECKFE peptide was synthesized using the standard Fmoc solid-phase method, then purified by RP-HPLC (Supplementary Figure S1A), the molecular weight was confirmed by MALDI-TOF MS (Supplementary Figure S1B). The CD spectrum (Supplementary Figure S1C) and TEM image (Supplementary Figure S1D) showed that the formation of peptide nanofibers with *β*-sheet structure. Subsequently, 1-(2-hydroxyethyl)-3,3-dimethylindolino-6′-nitrobenzopyrylospiran was reacted with bromoacetyl bromide to obtain bromoacetyl spiropyran (Supplementary Figure S2A). The nucleophilic substitution of the thiol group of the FKFECKFE peptide to bromoacetyl spiropyran in the presence of DIPEA afforded the SP-modified peptide, FKFEC^SP^KFE (Supplementary Figure S2B), which was purified by RP-HPLC (Supplementary Figure S3A) and confirmed by MALDI-TOF MS (Supplementary Figure S3B).

### Photoisomerization of FKFEC^SP/MC^KFE peptides

**FIGURE 2:**
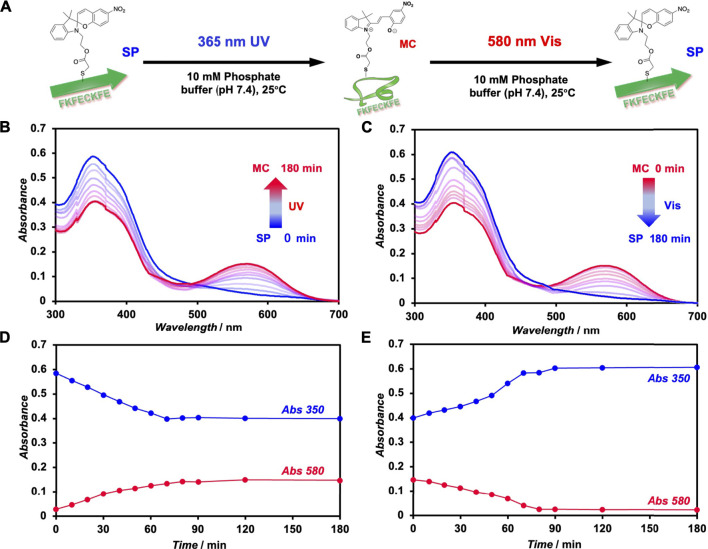
**(A)** Schematic of the photoisomerization of FKFEC^SP/MC^KFE peptides. UV–visible spectra of **(B)** spiropyran (SP)-modified peptide ([FKFEC^SP^KFE] = 100 μM, blue) during UV light (365 nm, 5 mW, 5 cm) irradiation and **(C)** merocyanine (MC)-modified peptide ([FKFEC^MC^KFE] = 100 μM, red) in a 10-mM phosphate buffer (pH, 7.4; 25°C) during visible light (580 nm, 5 mW, 5 cm) irradiation. Time course of absorbance of the SP (350 nm) and MC (580 nm) peptides during **(D)** UV- and **(E)** visible light irradiation.

The photoisomerization of FKFEC^SP^KFE peptides in a 10-mM phosphate buffer (pH, 7.4) was confirmed by UV–visible spectroscopy (Figure 2). An aqueous solution of 100 μM FKFEC^SP^KFE in a 10-mM phosphate buffer (pH, 7.4) was incubated at 60°C for 30 min, followed by UV light irradiation at 365 nm (80 min, 5 mW, 5 cm). The absorbance of SP (around 350 nm) in the peptide decreased, whereas that of MC (around 580 nm) increased upon UV irradiation (Figure 2B). Contrarily, after UV light irradiation, the peptide solution was irradiated with visible light at 580 nm (80 min, 5 mW, 5 cm), which increased the absorbance of SP and decreased the absorption of MC (Figure 2C). The time course of absorbance of the FKFEC^SP/MC^KFE peptides due to photoisomerization reached a steady state at 80 min for both UV- and visible light irradiation (Figures 2D, E). These results indicated that the FKFEC^SP/MC^KFE peptides were reversibly photoisomerized at approximately 80 min. RP-HPLC confirmed that the peptides were 91.4% FKFEC^MC^KFE and 8.6% FKFEC^SP^KFE after 80 min of UV light irradiation at 365 nm (Supplementary Figure S4; Supplementary Table S1).

### Secondary structure of FKFEC^SP/MC^KFE peptides

**FIGURE 3:**
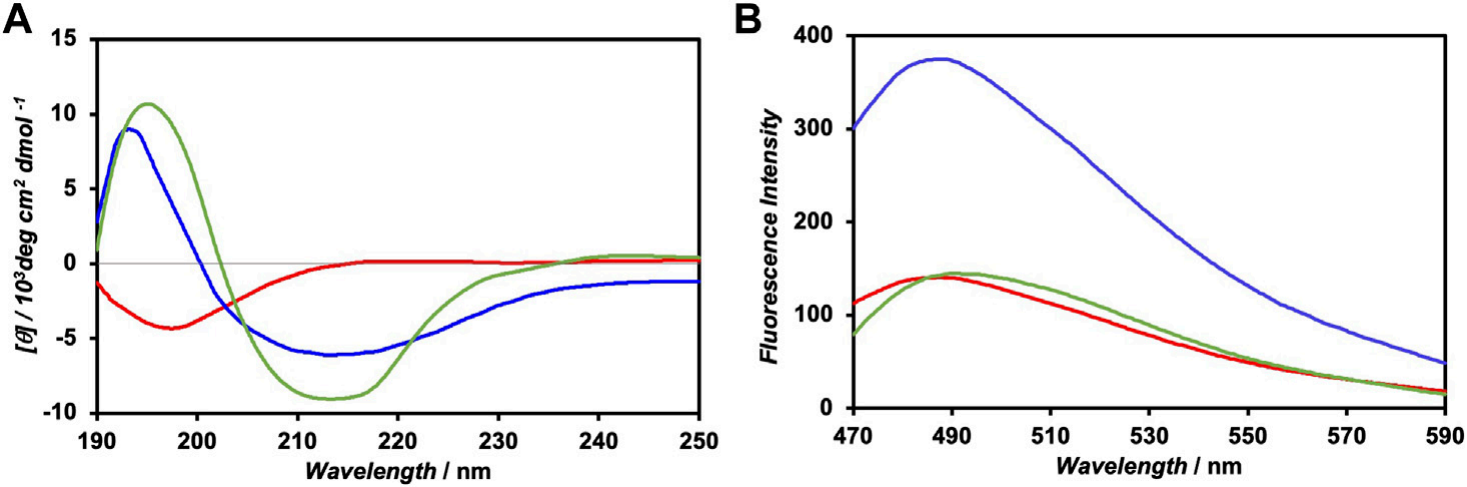
**(A)** CD spectra of 100-μM FKFEC^SP^KFE (blue), 100-μM FKFEC^MC^KFE (red), and FKFEC^SP^KFE resulted by 580 nm visible light irradiation to 100-μM FKFEC^MC^KFE and subsequent incubation in the dark for 24 h (green) in a 10-mM phosphate buffer (pH, 7.4) at 25°C. **(B)** Fluorescence spectra of thioflavin T (100 μM) in the presence of 100-μM FKFEC^SP^KFE (blue), 100-μM FKFEC^MC^KFE (red), and thioflavin T alone (green) in a 10-mM phosphate buffer (pH 7.4) at 25°C.

The CD spectrum of FKFEC^SP^KFE peptides in a 10-mM phosphate buffer (pH 7.4), which was incubated at 60°C for 30 min and left in the dark for 24 h at 25°C, exhibited positive and negative peaks at 197 and 214 nm, respectively, indicating the formation of a typical *β*-sheet structure (Figure 3A). Contrarily, the CD spectrum of the photoisomerized FKFEC^MC^KFE peptides in the same buffer exhibited a negative peak at 198 nm, indicating a random coil structure. After irradiation of visible light at 580 nm (80 min, 5 mW, 5 cm) to the FKFEC^MC^KFE peptide and subsequent incubation in the dark for 24 h at 25°C, the CD spectrum indicated the reformation of *β*-sheet structure. As most of the peptides reverted to the *β*-sheet conformation, the reversibility of the photoisomerization process of this peptide was confirmed. However, the secondary structure of the peptide was slightly different from that before photoisomerization, which is probably due to the generation of small amount of other structures such as *α*-helix after photoisomerization.

The secondary structure of the FKFEC^SP/MC^KFE peptides was also confirmed by the fluorescence spectra of thioflavin T; the fluorescence intensity increased when bound to nanofibers comprising *β*-sheet structures. The fluorescence intensity of thioflavin T in the presence of FKFEC^SP^KFE increased approximately three-fold, compared with that of thioflavin T alone, whereas FKFEC^MC^KFE minimally affected the intensity of thioflavin T (Figure 3B). These results suggested that FKFEC^SP^KFE peptides formed nanofibers with a *β*-sheet structure, whereas FKFEC^MC^KFE peptides exhibited a random coil structure.

### Polymerization and depolymerization of nanofibers controlled by the photoisomerization of FKFEC^SP/MC^KFE peptides

**FIGURE 4:**
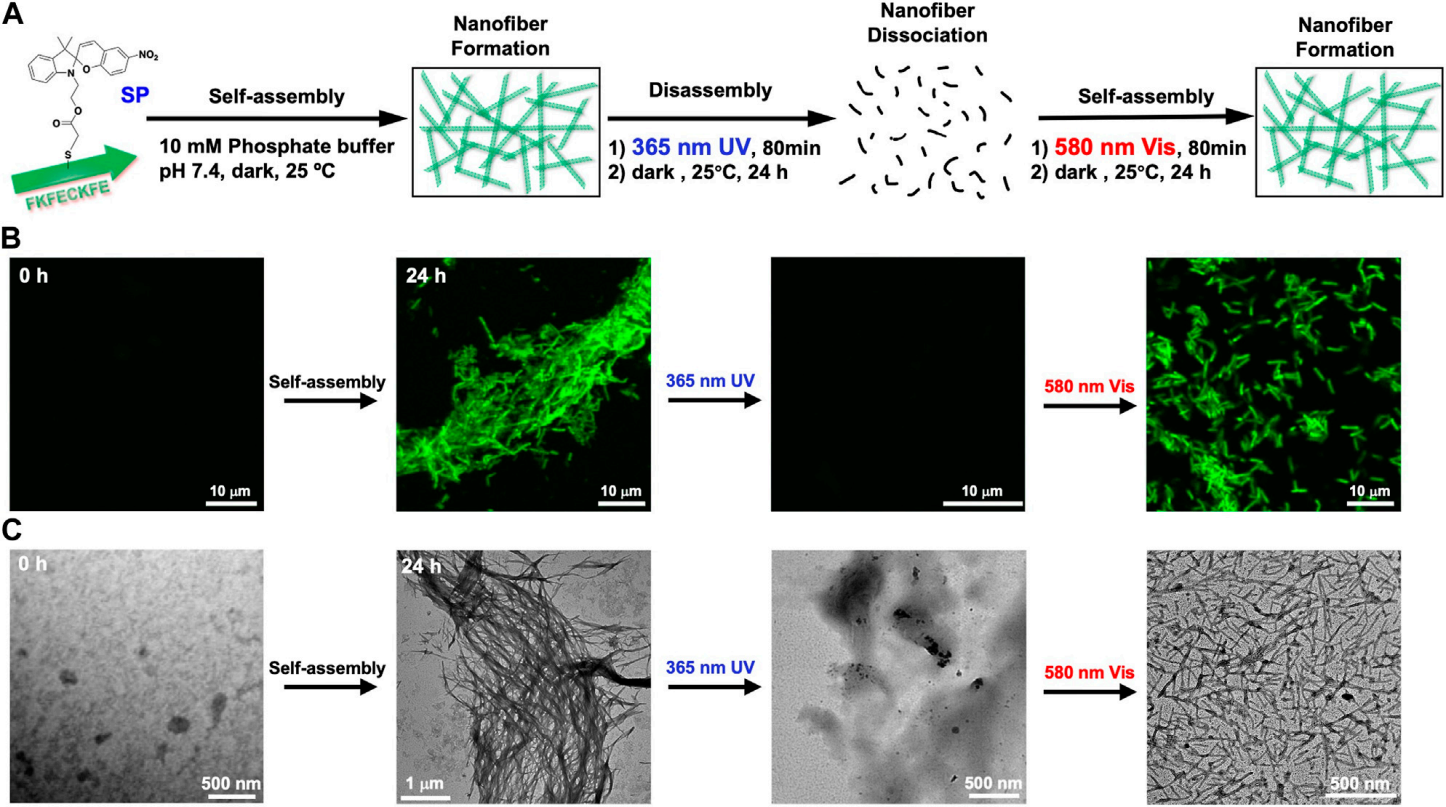
**(A)** Schematic of the formation and dissociation of the peptide nanofiber by photoisomerization. CLSM **(B)** and TEM images **(C)** of 100-μM FKFEC^SP/MC^KFE and 100 μM thioflavin T during photoisomerization to form and dissociate peptide nanofibers in a 10-mM phosphate buffer (pH, 7.4) at 25°C.

The change in self-assembling behavior driven by the photoisomerization of FKFEC^SP/MC^KFE peptides was evaluated by CLSM with thioflavin T staining and TEM with phosphotungstic acid staining (Figure 4A). No nanofiber images were observed immediately after the FKFEC^SP^KFE peptide solution was incubated at 60°C for 30 min and left in the dark at 25°C. After the incubation of the solution for 24 h, the formation of peptide nanofibers with lengths in the range of 1–7 μm was observed by CLSM and TEM, as shown in Figures 4B, C, respectively. After the FKFEC^SP^KFE peptide nanofibers were irradiated with UV light at 365 nm (80 min, 5 mW, 5 cm), followed by incubation in the dark for 24 h, the nanofibers completely disappeared (Figures 4B, C). This indicated that the structural change from *β*-sheets to random coils caused by the isomerization of SP to MC was responsible for the dissociation of the nanofibers. The reformation of nanofibers with lengths in the range of 0.6–3.5 μm was achieved when the FKFEC^MC^KFE solution was irradiated with visible light at 580 nm (80 min, 5 mW, 5 cm), followed by incubation in the dark for 24 h (Figures 4B, C). These results showed that the photoisomerization of FKFEC^SP/MC^KFE can reversibly control the formation and dissociation of nanofibers. It is possible that the initial heating step at 60°C to promote rapid dissolution of the powdered peptide in phosphate buffer provided the necessary energy to form longer fibers more rapidly. The shorter fibers observed in the second round of photoisomerization may be formed because there is no energy boost. In addition, the secondary structural change due to photoisomerization was not completely reversible (Figure 3A), which may be one of the factors contributing to the shorter length of the peptide nanofibers.

### Morphological changes in the GUV by the photoisomerization of FKFEC^SP/MC^KFE peptides

**FIGURE 5:**
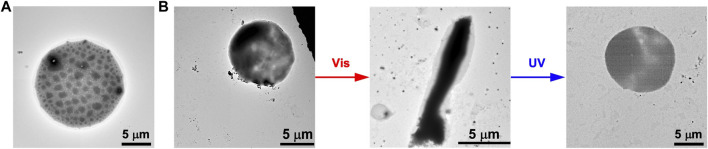
TEM images of **(A)** the GUVs alone and **(B)** the morphological change in the GUVs ([POPC] = 1 mM) by the photoisomerization of FKFEC^SP/MC^KFE peptides in a 10-mM phosphate buffer (pH, 7.4) at 25°C.

Furthermore, we evaluated if the photoisomerization of FKFEC^SP/MC^KFE can transform the morphology of spherical GUVs. A mixed lipid membrane of POPC and Atto 550-labeled DOPE was hydrated to form spherical GUVs (Figure 5A). Also, the mixed lipid membrane was hydrated with an aqueous solution of random coil FKFEC^MC^KFE peptide to encapsulate the peptide with GUVs. The TEM image of the complex of FKFEC^MC^KFE peptides and POPC shows the formation of normal spherical GUVs (Figure 5B). After visible light irradiation at 580 nm (80 min, 5 mW, 5 cm), the spherical GUV was significantly deformed to a rod-like structure. When this sample solution was irradiated with UV light at 365 nm (80 min, 5 mW, 5 cm), spherical GUVs were again observed (Figure 5B).

**FIGURE 6:**
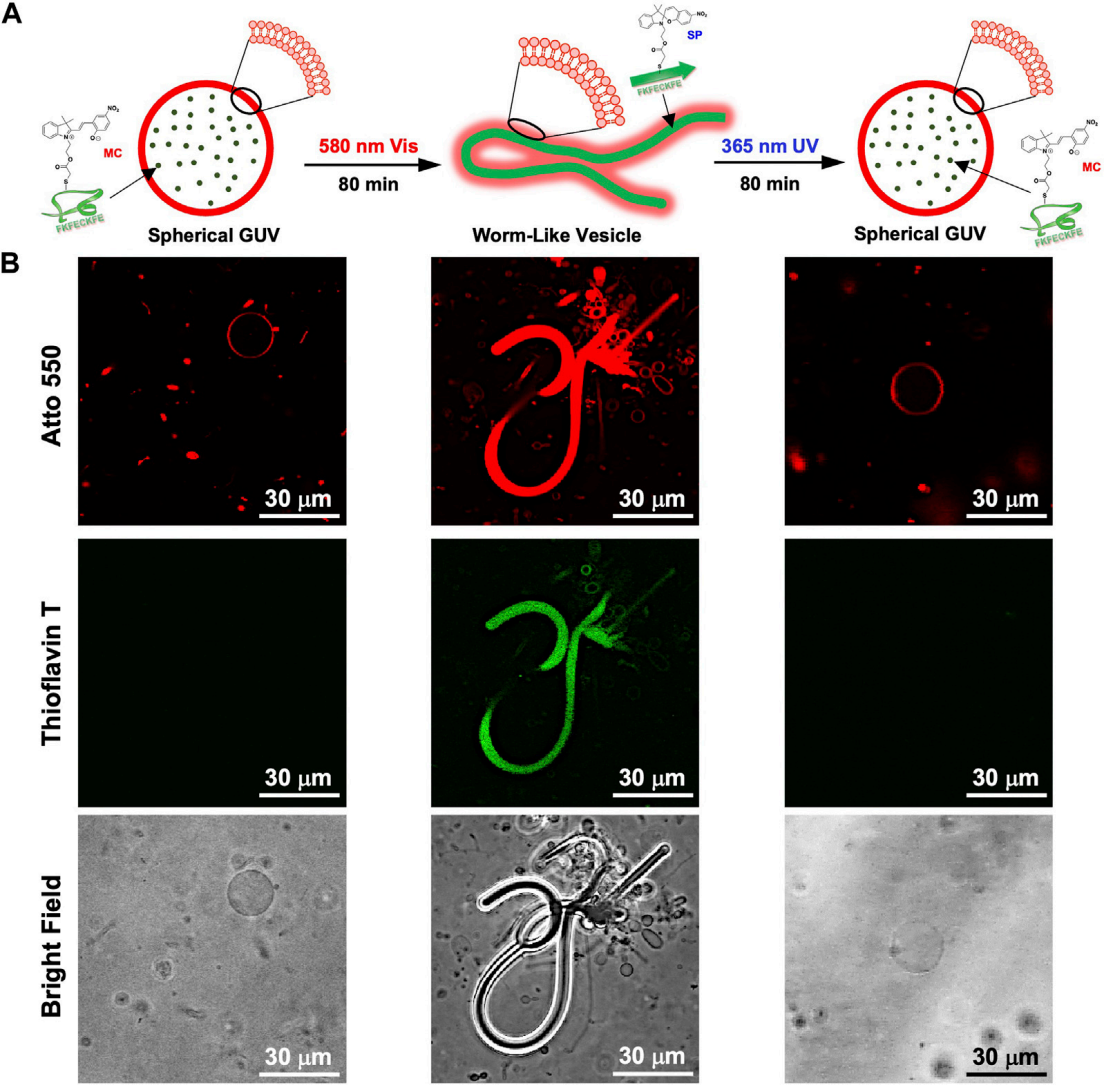
**(A)** Schematic of the morphological change in the GUV by the photoisomerization of peptides. **(B)** CLSM images of the morphological change in the GUV ([Atto 550-DOPE] = 5 μM [POPC] = 1 mM) encapsulated using 10-μM FKFEC^MC^KFE and 100 μM thioflavin T by photoisomerization in a 10-mM phosphate buffer (pH, 7.4) at 25°C. Channel for Atto 550 (top), thioflavin T (middle), bright field (bottom).

**FIGURE 7:**
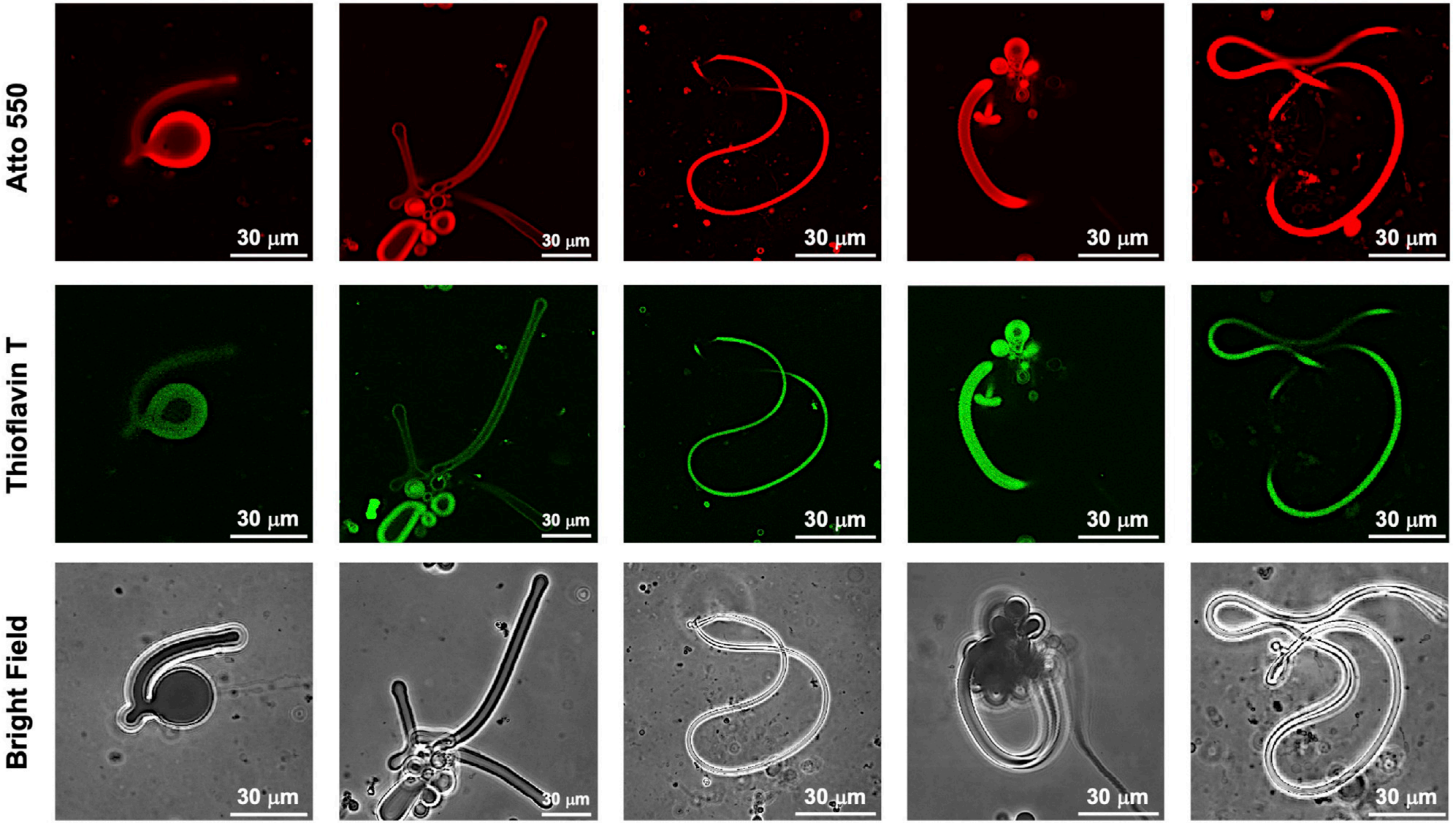
CLSM images of various morphologies of worm-like vesicles ([Atto 550-DOPE)] = 5 μM [POPC] = 1 mM) encapsulated using 10-μM FKFEC^SP^KFE and 100 μM Thioflavin T in a 10-mM phosphate buffer (pH, 7.4) at 25°C. Channels for Atto 550 (top), thioflavin T (middle), and bright field (bottom).

While dry samples were observed by TEM, CLSM was used to observe in aqueous solution the morphological changes in the GUVs due to the photoisomerization of FKFEC^MC^KFE peptide by thioflavin T staining (Figure 6A). The GUV composed of POPC/Atto-550 POPE encapsulating FKFEC^MC^KFE peptide exhibited a normal spherical morphology in the aqueous solution. Interestingly, visible light irradiation at 580 nm (80 min, 5 mW, 5 cm) induced a dramatic morphological change into a worm-like vesicle (Figure 6B; after 505 nm, 52 mW, 10 cm, 3 min irradiated, Supplementary Video S1). This change was probably caused by the formation of peptide nanofibers inside the GUV by photoisomerization from MC to SP. Visible light irradiation of the GUV encapsulating FKFEC^MC^KFE peptide induced a variety of morphologies of worm-like vesicles, and an intermediate state of worms extending from inside the spherical GUVs was also observed (Figure 7). The worm-like vesicle completely reverted to the original spherical GUV after UV light irradiation at 365 nm (after 80 min, 5 mW, 5 cm, Figure 6B; and after 30 mW, 10 cm, 3 min irradiated, Supplementary Video S2). Although the photoisomerization of the peptide at 10 μM does not cause bursting, the peptide could not be encapsulated by GUV when higher peptide concentrations (50 and 100 μM) were mixed with GUV. Furthermore, the photoisomerization of the peptide at 5 μM induced small morphological changes in the GUV, and at 1 μM did not induced morphological changes (Supplementary Figure S9).

The presence of the FKFEC^SP^KFE peptide inside the worm-like vesicle was confirmed by the fluorescence profile in the CLSM image (Supplementary Figure S5A). The fluorescence profile of thioflavin T can be observed inside that of Atto 550 on the white line in the merged image, suggesting that the FKFEC^SP^KFE peptide was internalized within the GUV and partially adsorbed in the membrane (Supplementary Figure S5B). Since merocyanine dye itself fluoresces at 577 nm when excited at 553 nm, the CLSM of the unstained FKFEC^MC^KFE peptide-GUV mixture revealed MC-derived red fluorescence within the GUV (Supplementary Figure S6A). The fluorescence profiles revealed that the FKFEC^MC^KFE peptide was localized within the GUV and partially adsorbed on the membrane (Supplementary Figure S6B).

## Discussion

Dynamics of cytoskeleton are important for deformation and movement of various membrane systems in the cell, arrangement of cell organelles, cell division, muscle contraction, and ciliary motility. Mimicking the cytoskeleton dynamics, we developed a short peptide FKFEC^SP/MC^KFE which can be reversibly polymerized and depolymerized by the photoisomerization. In addition, dramatic and reversible morphological change of cell model GUV encapsulating FKFEC^MC^KFE was induced by the photoisomerization.

Although several studies have been reported on changing the morphology of GUVs in various ways, there were some problems such as irreversibility (Loiseau et al., 2016), long response time (Fanalista et al., 2018), and small morphology change (Li et al., 2021). In this study, dramatic and reversible morphology change of GUVs was achieved by brief photoisomerization of FKFEC^SP/MC^KFE peptide inside the GUV using a strong light source. This morphology change is attributed to the formation of the *β*-sheet peptide nanofibers inside GUV due to the large structural change from MC to SP upon visible light irradiation. The polymerization of the numerous peptides accompanied by the lipid bilayer-peptide interaction inside GUV could enhance extrusion of the membrane outward to form worm-like vesicles (Figure 6B; Supplementary Video S1). The worm-like vesicles were reverted to the spherical morphology by UV light irradiation (Figure 6B; Supplementary Video S2). The relaxation of deformation stress could be caused by the depolymerization of peptide nanofibers inside vesicle due to photoisomerization from SP to MC.

In this study, we succeeded in reversibly changing the morphology of GUVs by photoisomerization of nanofiber-forming peptides. The molecular design mimicking the polymerization and depolymerization of the cytoskeleton provides a guideline for a photo-controlled artificial cytoskeleton and can be used as a component of molecular robots and artificial cell systems (Hagiya, et al., 2014; Murata, et al., 2022). However, the present study has shown that construction of artificial cytoskeletons is hindered by the relatively slow photoisomerization process compared to the fast time scale of the natural cytoskeleton. To overcome this limitation, the design of nanofiber-forming peptide could be optimized to allow faster assembly and disassembly kinetics. Alternatively, the use of alternative approaches, such as light-activated proteins or other molecular switches, could allow more precise and rapid control of cytoskeletal deformation. In the future, we are planning to actually introduce the photo-controlled artificial cytoskeleton into cells to control cell deformation and migration by photoisomerization. We will introduce the peptide fibers into cells to evaluate their effects on light-induced migration, thereby providing of the possibility of light-driven artificial cytoskeletons for manipulation of cellular structures.

## Conclusion

We synthesized a FKFEC^SP/MC^KFE peptide in which the central Phe residue of the *β*-sheet structure-forming peptide FKFEFKFE was replaced by a Cys residue modified with a photoisomerizable SP/MC. The photoisomerization ability and the secondary structure were confirmed by UV-Vis spectra and CD spectra. Reversible control of polymerization and depolymerization of nanofibers was achieved by photoisomerization of the FKFEC^SP/MC^KFE peptide. Interestingly, the visible light irradiation of the spherical GUV-encapsulating FKFEC^MC^KFE peptide induced a dramatic morphological change that the spherical vesicles transformed from spherical to worm-like vesicles. Polymerization and depolymerization of peptide nanofibers by photoisomerization in the vicinity of the lipid bilayer seems to promote extrusion and relaxation of the membrane. This molecular design provides guidelines for light-controlled artificial cytoskeletons that mimic the polymerization/depolymerization of the cytoskeleton. We envisage that light-controlled artificial cytoskeletons can be introduced into cells, which will be an innovative molecular technology to control cellular deformation and migration by light.

## Data Availability

The original contributions presented in the study are included in the article/; Supplementary Material, further inquiries can be directed to the corresponding author.
