# Supplementary material for: Dramatic morphological changes in liposomes induced by peptide nanofibers reversibly polymerized and depolymerized by the photoisomerization of spiropyran
Source: Front Mol Biosci. 2023 Mar 30;10:1137885. doi: 10.3389/fmolb.2023.1137885 (PMC10101338; doi:10.3389/fmolb.2023.1137885)
Supplement: Supplementary file 1 [file DataSheet1.pdf]

Supplementary Material

Dramatic Morphological Changes in Liposomes induced by Peptide Nanofibers Reversibly Polymerized and Depolymerized by the Photoisomerization of Spiropyran

Yingbing Liang, Shigesaburo Ogawa, Hiroshi Inaba, Kazunori Matsuura*

*** Correspondence:** Kazunori Matsuura: ma2ra-k@tottori-u.ac.jp

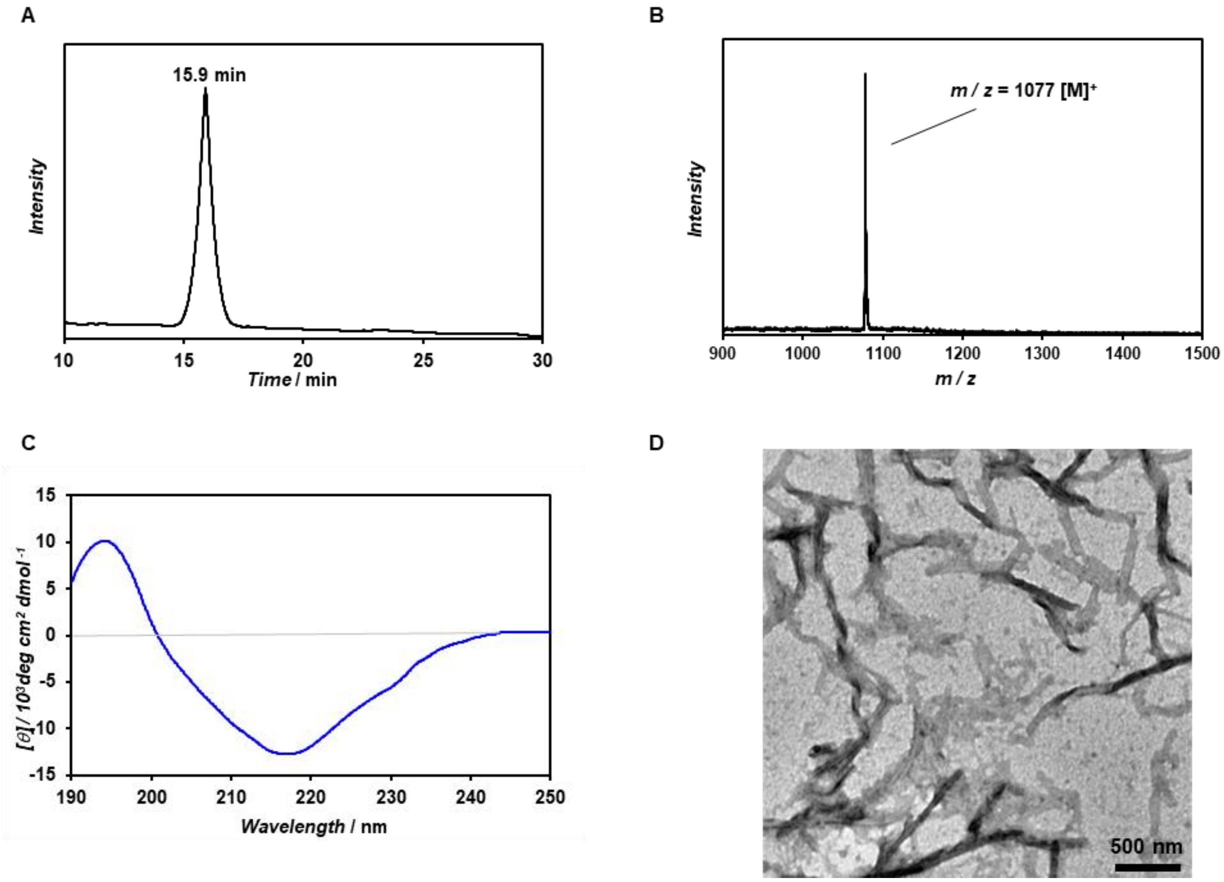

Figure S1. Reversed-phase HPLC chart **(A)** , MALDI-TOF-MS **(B)** of purified FKFECKFE peptide, (C) CD spectrum and (D) TEM image.

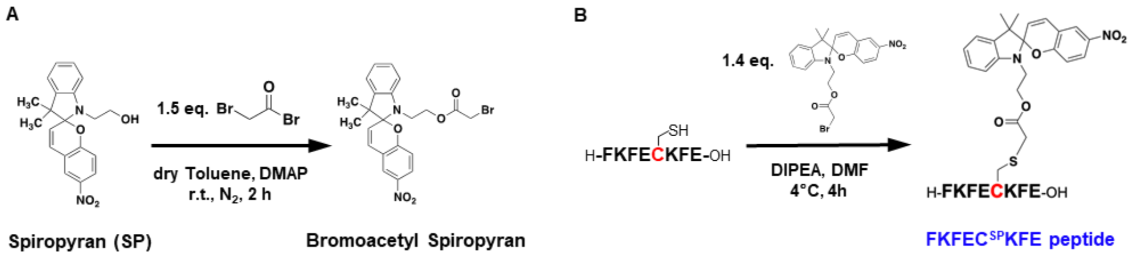

Figure S2. Synthesis of **(A)** bromoacetyl spiropyran, and **(B)** FKFEC^{SP}KFE peptide.

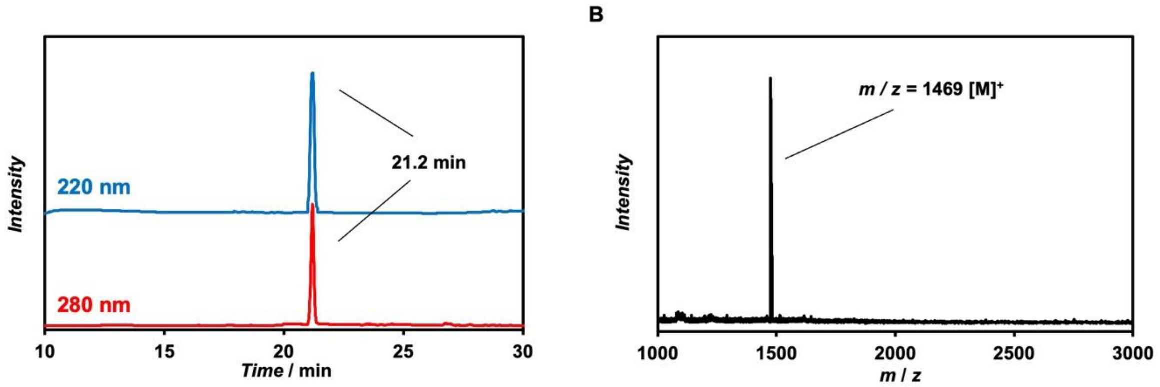

Figure S3. Reversed-phase HPLC chart **(A)** and MALDI-TOF-MS **(B)** of purified FKFEC^{SP}KFE peptide.

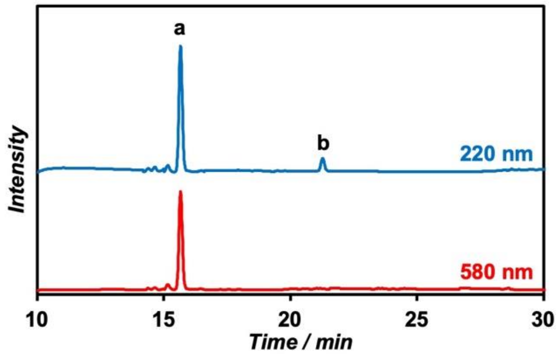

Figure S4. Reversed-phase HPLC chart of FKFEC$^{SP/MC}$KFE peptide after UV light irradiation for 80 min.

TABLE S1. Ratio of each peaks in Figure S4.

Peak	Peptide	Retention time	Area	Ratio
a	FKFEC^{MC}KFE	15.6 min	249633	91.4 %
b	FKFEC^{SP}KFE	21.3 min	23251	8.6%

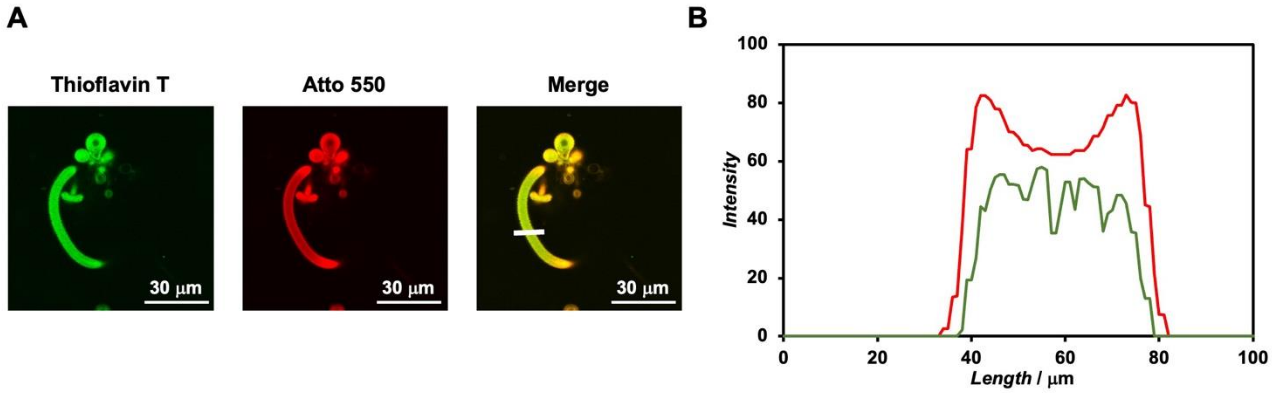

Figure S5. (A) CLSM images of worm-like vesicles ([Atto 550-DOPE] = 5 μM, [POPC] = 1 mM) encapsulating 10 μM FKFEC^{SP}KFE peptide and 100 μM thioflavin T in 10 mM phosphate buffer (pH 7.4) at 25°C. Channel for thioflavin T (left), Atto 550 (middle), merge (right). **(B)** Fluorescence profile of thioflavin T (green) and Atto 550 (red) on the white line in the merge image.

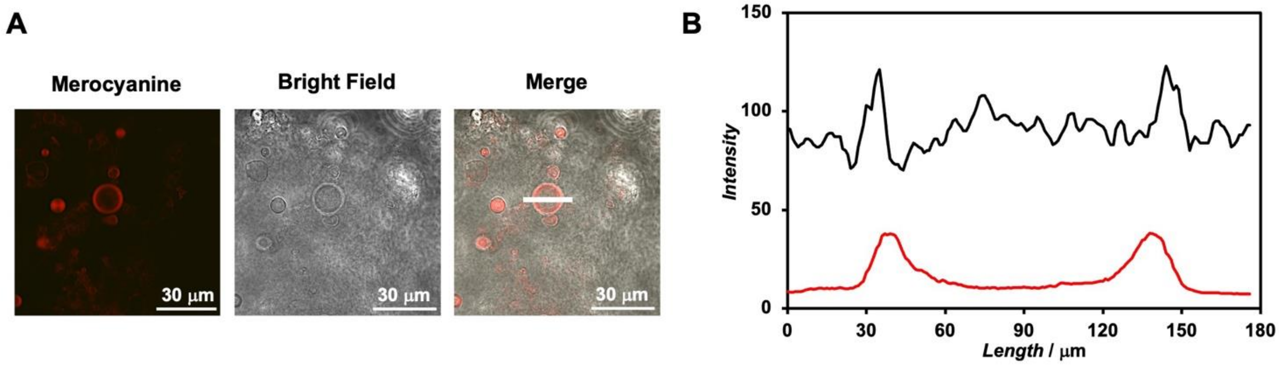

Figure S6. (A) CLSM images of GUVs ([POPC] = 1 mM) encapsulating 10 mM FKFEC^{MC}KFE peptide in 10mM phosphate buffer (pH 7.4) at 25°C. Channel for merocyanine (left, Ex: 553nm, Em: 577 nm), bright field (middle), merge (right). **(B)** Fluorescence profile of merocyanine (red) and bright field (black) on the white line in the merge image.

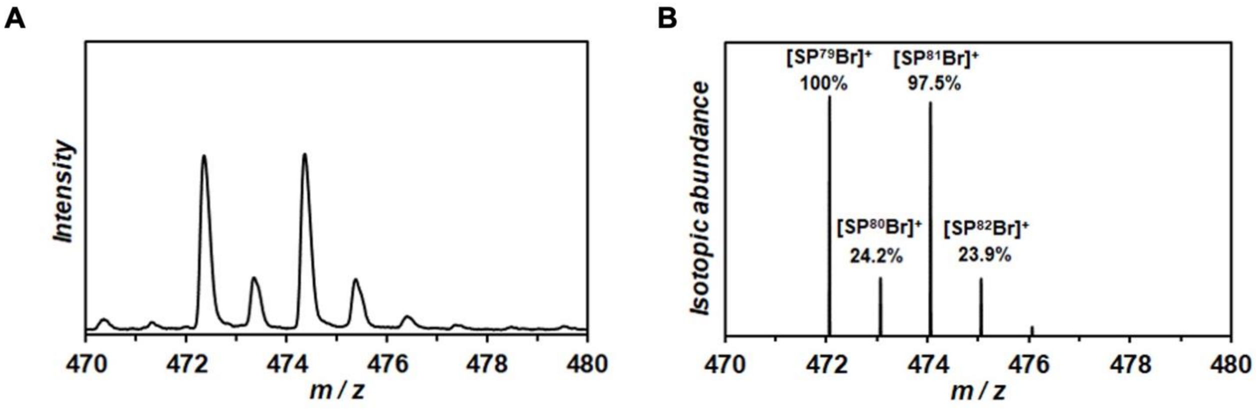

Figure S7. (A) MALDI-TOF-MS of bromoacetyl spiropyran, **(B)** Percentage of $[SP\text{-}Br]^+$ calculated from ChemDraw.

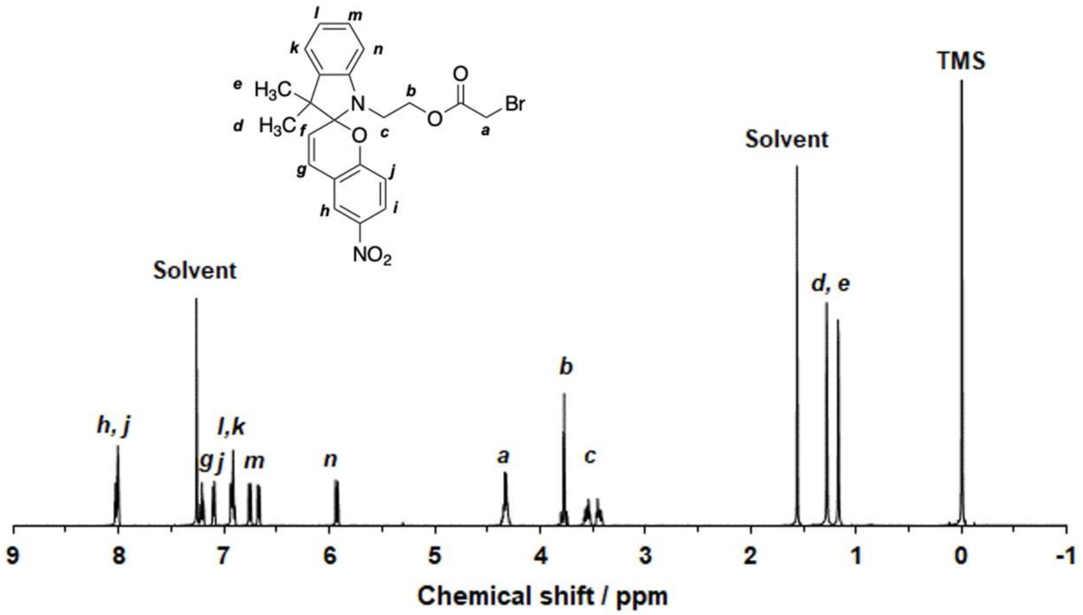

Figure S8. ^{1}H-NMR spectrum ($CDCl_3$) of bromoacetyl spiropyran.

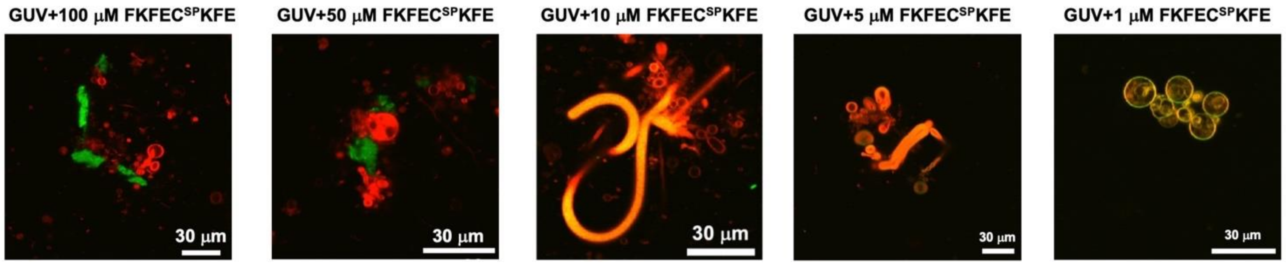

FIGURE S9. Concentration dependence of FKFEC^{MC}KFE peptide on the morphological change of the GUV ([Atto 550 DOPE] = 5 μM, [POPC] = 1 mM) by photoisomerization. Various concentrations of FKFEC^{MC}KFE peptide (100 μM, 50 μM, 10 μM, 5 μM, and 1 μM) and 100 μM thioflavin T was mixed with the GUV and then visible light at 580 nm was irradiated for 80 min (5 cm, 5 mW) in 10 mM phosphate buffer at 25ºC. Five CLSM images are shown using a merge channel of Atto 550 and Thioflavin T.
